# Literary Notes

**Published:** 1910-01

**Authors:** 


### LITERARY NOTES

THE *Annals of Surgery* for December, 1909, is a jubilee edition. It is a pretentious volume of 400 pages which is a credit to medical journalism and the editor and staff are to be congratulated upon the production of a magazine of such magnitude and excellence. The articles are nearly all written by leading authorities and many are well illustrated. The general arrangement and make up is of the best. The *Annals of Surgery* well fills a leading place in surgical journalism.

"MEDICAL LIBRARIES" is the title of a brochure reprinted from the *Medical Record,* September 25, 1909, prepared by the librarian of the New York Academy of Medicine. It contains a list of the medical libraries throughout the civilised world, giving the post office, name of the librarian and number of bound volumes in each. Collections of medical works in general libraries are not given. It is a useful list for reference and corrections as well as inquiries. Further information can be had by addressing Mr. John S. Browne, librarian, New York Academy of Medicine, 17 W. 43d Street, New York.

